# Atlantic deep water circulation during the last interglacial

**DOI:** 10.1038/s41598-018-22534-z

**Published:** 2018-03-13

**Authors:** Yiming Luo, Jerry Tjiputra, Chuncheng Guo, Zhongshi Zhang, Jörg Lippold

**Affiliations:** 1 Geophysical Institute, University of Bergen and Bjerknes Centre for Climate Research, Allegaten 70, 5007 Bergen, Norway; 2 Uni Research Climate, and Bjerknes Centre for Climate Research, Jahnebakken 5, 5007 Bergen, Norway; 3 Institute of Earth Sciences, Heidelberg University, Im Neuenheimer Feld 234, 69120 Heidelberg, Germany

## Abstract

Understanding how the Atlantic Meridional Overturning Circulation (AMOC) evolved during crucial past geological periods is important in order to decipher the interplay between ocean dynamics and global climate change. Previous research, based on geological proxies, has provided invaluable insights into past AMOC changes. However, the causes of the changes in water mass distributions in the Atlantic during different periods remain mostly elusive. Using a state-of-the-art Earth system model, we show that the bulk of NCW in the deep South Atlantic Ocean below 4000 m migrated from the western basins at 125 ka to the eastern basins at 115 ka, though the AMOC strength is only slightly reduced. These changes are consistent with proxy records, and it is mainly due to more penetration of the AABW at depth at 115 ka, as a result of a larger density of AABW formed at 115 ka. Our results show that depth changes in regional deep water pathways can result in large local changes, while the overall AMOC structure hardly changes. Future research should thus be careful when interpreting single proxy records in terms of large-scale AMOC changes, and considering variability of water-mass distributions on sub-basin scale would give more comprehensive interpretations of sediment records.

## Introduction

Oceanic circulation is one of the most important driving forces of the climate system, because it redistributes both heat and carbon in the ocean interior^1^ and hence influences atmospheric CO_2_ levels. As such, changes in the deep-water circulation, especially in the Atlantic Ocean, are of particular interest in order to understand past and future climate changes. Identifying and understanding the relationship on different time scales between past climate variations and circulation changes has therefore been pursued by oceanographers and climate scientists for decades^2–8^.

Multiple chemical proxies, including both stable isotopes^9^ (e.g., benthic δ^13^C) and radiogenic isotopes^10^ (e.g., ε_Nd_ and ^231^Pa/^230^Th), have been developed over the last few decades^11^ for this purpose. For instance, several studies, which include sediment records from the South Atlantic (SA), suggest that the overturning circulation reversed during some critical geological periods^6^. In particular, during the Last Glacial Maximum (LGM), the ocean circulation structure underwent a massive re-organization^12–14^. Considering the SA’s key role as an active passageway connecting and regulating the North Atlantic (NA) and the Southern Ocean (SO) deepwater masses, rather than simply acting as a passive conduit^15^, a better understanding of how the SA water-mass distributions evolved during such periods is important to improve our knowledge of the connection between the climate change and the variations of deep water circulation.

An informative analogue to future climate conditions would be the last interglacial period, spanning the period ~130–115 ka (BP). This time interval corresponds to Marine Isotope Stage (MIS) 5e^16^, which begins with glacial termination, initiated before 130 ka, reaches through the interglacial climatic optimum at about 128 ka^17^, and cools afterwards to approach glacial inception after 115 ka^18,19^.

Here, we examine the spatial structure and variations of AMOC at two time periods during the last interglacial (115 ka and 125 ka) through analyzing quasi-equilibrium simulations of a state-of-the-art Norwegian Earth System Model^20^ (NorESM, see method for details). The simulations are based on widely-recognized, well-developed boundary climatic conditions (see Table S1 in SI)^21^. Through comparison of our model projections with available paleo-proxies, we aim to explain how changes in the deep water properties in the two periods affect the water mass distributions in the interior Atlantic Ocean.

## AMOC during the last interglacial

**Figure 1:**
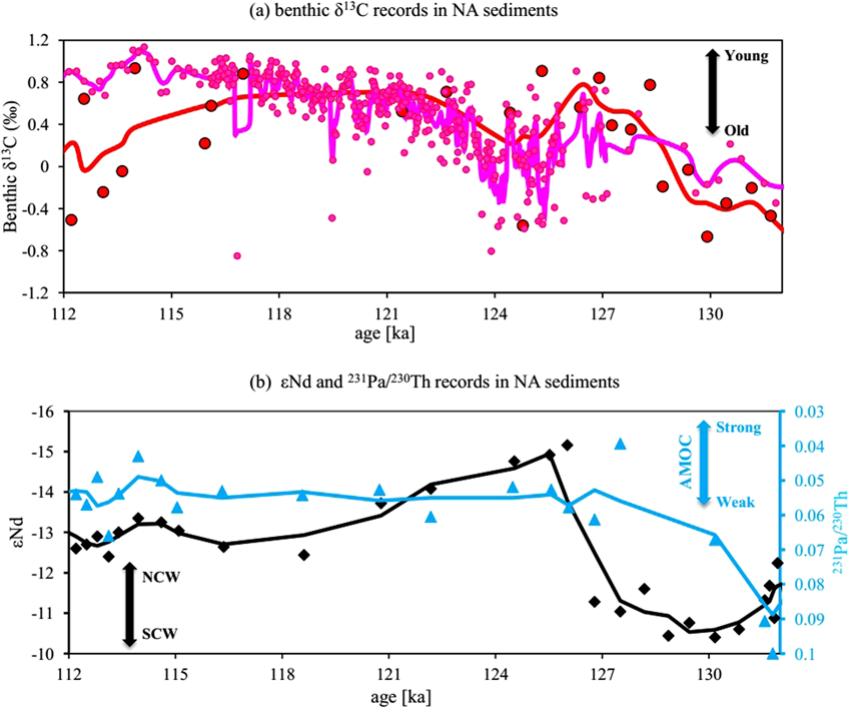
Proxies of past circulation from the NA: (**a**) benthic δ^13^C at ODP Site 1063 (red filled circles; 33.69°N, 57.62°W, 4,584 m water depth)^26^ and MD03-2664 (pink dots; 57.44°N, 48.61°W; 3,442 m water depth)^24^; (**b**) sedimentary εNd (blue triangle) and ^231^Pa/^230^Th (black diamonds) from ODP Site^22^ 1063. Curves show the three-point running average of the data with the corresponding color; proxy-records shown on their original chronology; locations of the two coring sites are indicated in Fig. 2.

The most compelling feature of the AMOC during the last interglacial, as derived from sediment records, is that the transition from the pre- to the late-interglacial conditions mirrors the behavior of the AMOC during the LGM and the deglacial to the late Holocene^13,22,23^ (Fig. 1). More specifically, multi-proxy records from the SA^13^ and the northwest Atlantic^22^ indicate a switch from Southern Component Water (SCW)-dominated water mass in the pre- last interglacial to Northern-Component-Water (NCW)-dominated water mass in the last interglacial.

Moreover, benthic δ^13^C from the northern NA (MD03-2664, 57.44°N, 48.61°W, 3,442 m water depth^24^; pink dots in Fig. 1a) quickly decreases from 0.4‰ (±0.3‰) at 127 ka to −0.05‰ (±0.52‰) at around 125 ka and then slowly increases to towards values around 0.8‰ (±0.14‰) at 115 ka. This has been used to suggest that AMOC experienced significant weakening at about 125 ka and gradual resumption afterwards^24,25^. A very similar trend is seen at ODP Site 1063 (33.69°N, 5762°W, 4,584 m water depth)^26^. Its benthic δ^13^C record decreases from 0.55‰ (±0.2‰) at 127 ka to 0.3‰ (±0.35‰) at around 125 ka and recover afterwards to reach 0.45‰ (±0.52‰) at 115 ka. Results from ODP Site 1063 have been used to suggest AMOC was relatively stable from 125 to115 ka^26^. The ε_Nd_ (−14.5 (±0.5) at around 125 ka versus −13.2 (±0.6) at around 115 ka) and ^231^Pa/^230^Th records (0.065 (±0.005) at around 125 ka versus 0.065 (±0.007) at around 115 ka) from the same location (ODP Site 1063) don’t change significantly over 125 to115 ka^22^ (Fig. 1). These results suggest a strong and persistent AMOC in this period^22^, consistent with the benthic δ^13^C record. Although we do not attempt to reconcile discrepancies between sedimentary records from different coring sites in the NA, our model output also implies a relatively constant (<2.5 Sv variations) strength of AMOC over the course of the last interglacial (Fig. S1 in SI).

**Figure 2:**
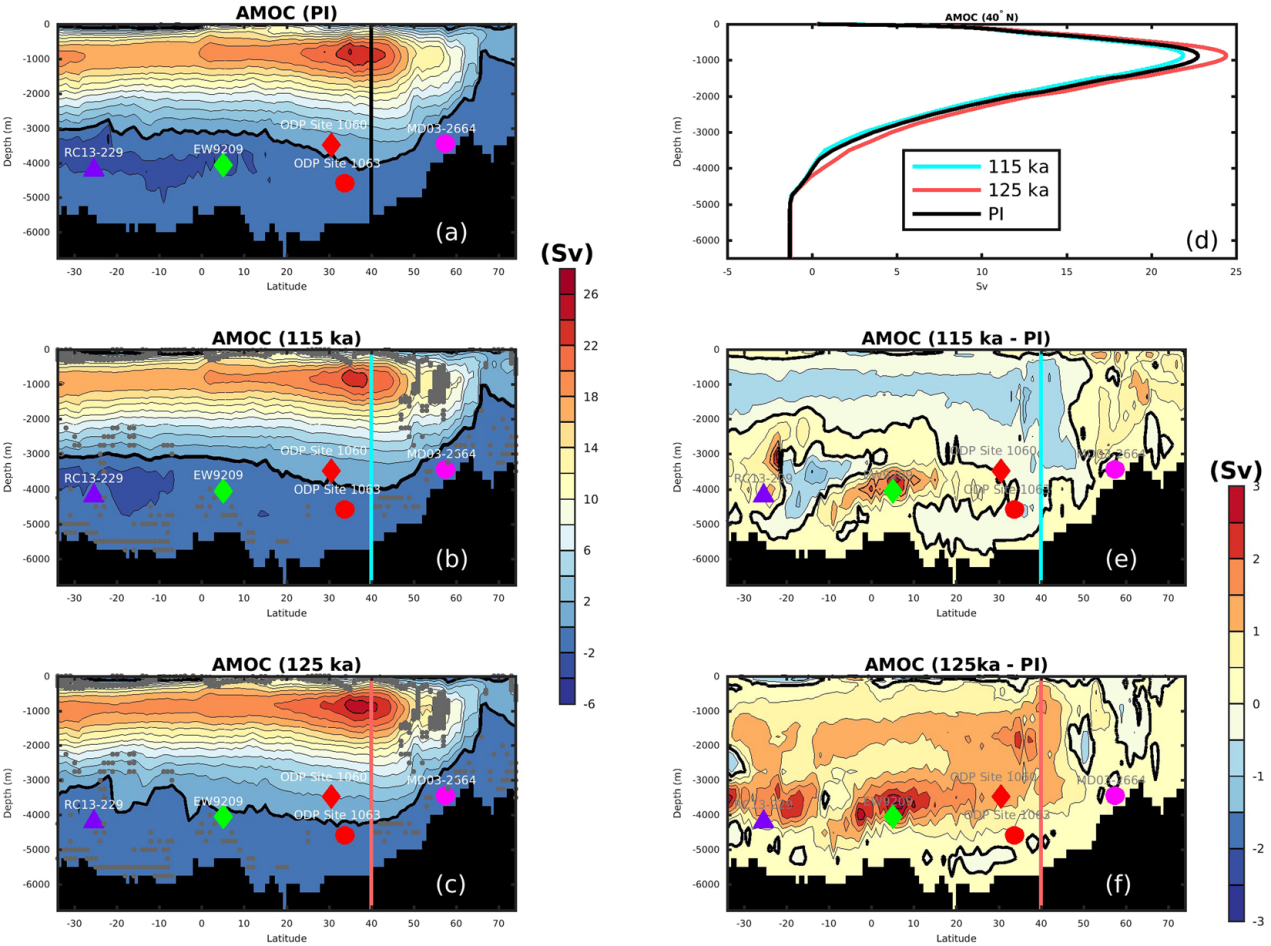
Simulated Atlantic Meridional Overturning Circulation (AMOC) for: (**a**) Pre-industrial/PI, (**b**) 115 ka and (**c**) 125 ka, (**d**) vertical profiles at 40°N for PI, 115 ka and 125 ka (see vertical line in a,b,c,e,f), (**e**) difference between 115 ka and PI, (**e**) difference between 125 ka and PI. Markers and core names indicate the coring locations of available δ^13^C records used in Figs 1 and 5. Thickened black contours indicate 0 Sv. Grey stippling in (**b**) and (**c**) indicate grids with statistically identical model results for 115 ka and 125 ka at the 5% significance level (h = 0 from the Student’s t-test, suggesting that the t-test does not reject the null hypothesis). Figure made with MATLAB R2014b.

Compared to the simulated AMOC during the preindustrial ages (PI), the AMOC at 115 ka was only about 0.5 Sv weaker and 100 m shallower while the AMOC at 125 ka was about 1.5 Sv stronger and 200 m deeper (Fig. 2). Simulated changes in the basin-wide AMOC structure between 125 ka and 115 ka are small, with the NADW about 2 Sv stronger and penetrating to a slightly greater (about 300 m deeper) depth at 125 ka (Fig. 2). As a result, the NCW appears to occupy a larger region of the deep meridional section of the Atlantic Ocean at 125 ka compared to the situation at 115 ka. This simulated feature is consistent with the ε_Nd_ records from the NA^22^, but appears to contrast with the interpretation of sediment proxy data from the SA, which suggests increased NCW in the SA at ~115 ka^13^. In order to clarify the link between the proxy records and the AMOC transition between 125 ka to 115 ka, a closer examination of the spatial and temporal water mass distributions in the SA is presented in this study.

## East-West water-mass distributions in the SA

Simulated latitudinal distributions of interior water masses in both the Eastern Atlantic (EA) and Western Atlantic (WA) are separated into NCW and SCW components using the “PO” tracer^27^. PO, which combines phosphate and oxygen concentrations corrected for biological respiration (PO = 172 × PO_4_ + O_2_), is a passive conservative water-mass tracer that has been widely used in water-mass studies to disentangle sources and properties of the interior ocean^28,29^. Here, it is used to distinguish northern- and southern-sourced water masses in the interior Atlantic, characterized by low (nutrient depleted) and high PO values, respectively.

**Figure 3:**
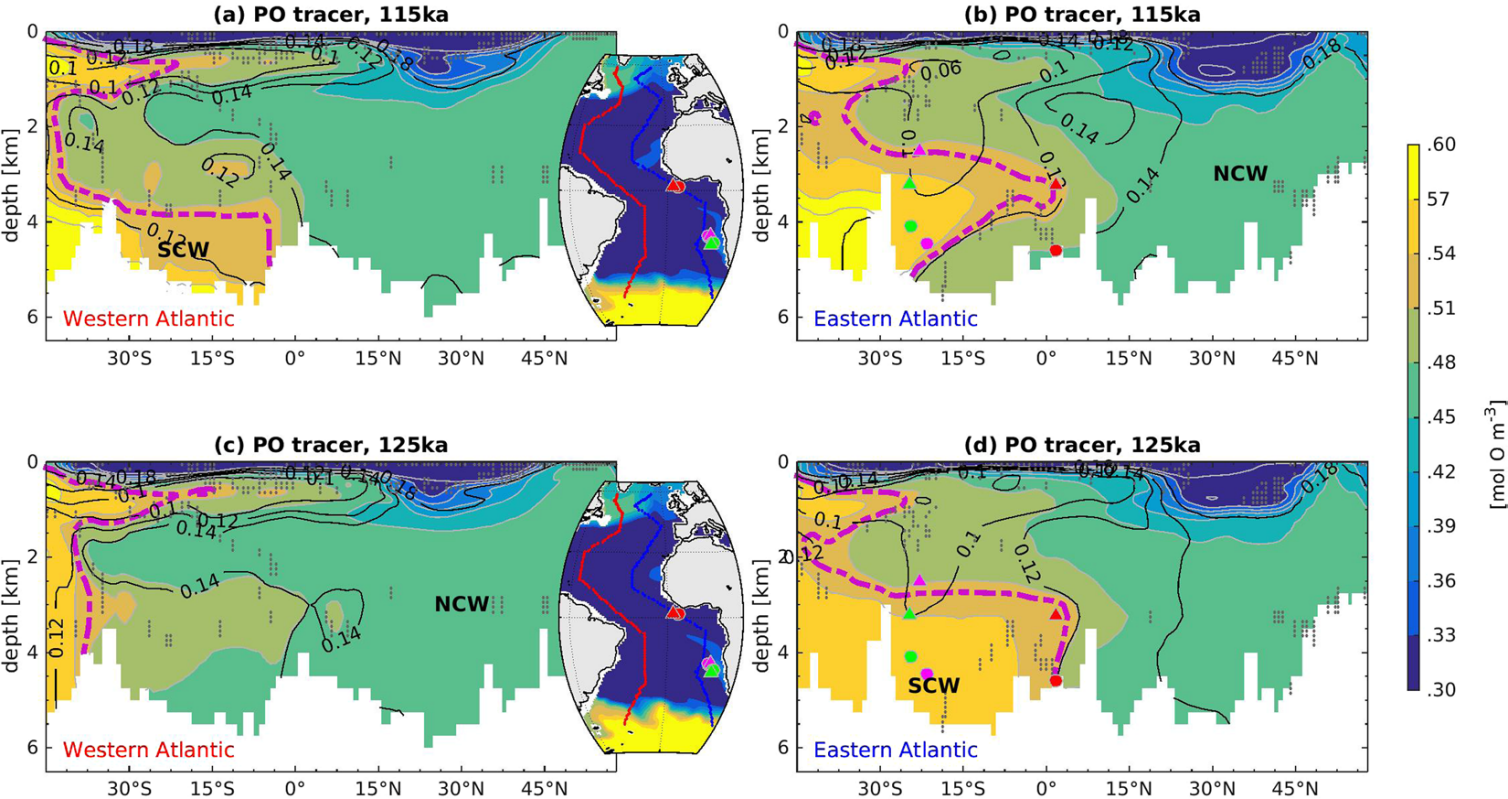
Water mass distributions indicated by PO tracer in the Atlantic Ocean, which show strong local water mass changes in the deep (below 4000 m) South Atlantic (south of 30°S). Shown are transects in the (**a**) western and (**b**) eastern Atlantic basin for the 115 ka. (**c**) and (**d**) show the same transects for the 125 ka. Contour lines with numbers depict the carbonate ion (CO_3_^2−^) concentration in [mol m^−3^] unit. Grids with grey stippling indicate areas where results for 115 ka and 125 ka are statistically identical (T-test does not reject the null hypothesis at the 5% significance level).Markers indicate locations of the sediment cores for sand content in Fig. 5. Pink dash lines show the approximate boundary between the NCW and SCW. Figure made with MATLAB R2014b.

**Figure 4:**
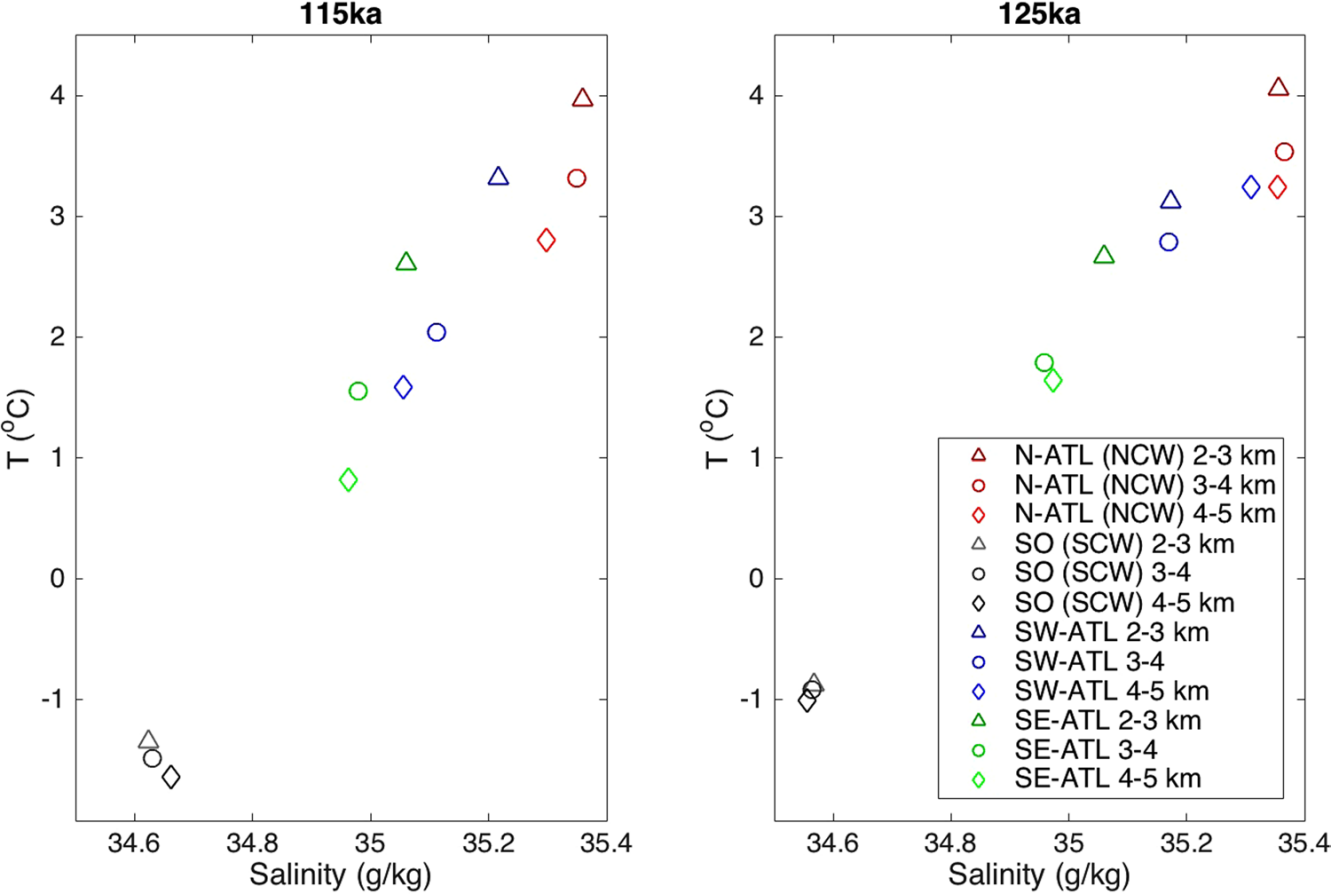
Temperature-Salinity plot for deep water below 2000 m in four different regions in the Atlantic Ocean at (**a**) 115 ka and (**b**) 125 ka. Data for different groups are taken from the average value of model simulations for: N-ATL (North Atlantic; Latitude: ~45–60°N, Longitude: ~45–20°W); SO (Southern Ocean; Latitude ~70–65°S, Longitude ~31–1°W); SW-ATL (Southwest Atlantic; Latitude ~15–40°S, Longitude ~35–25°W); SE-ATL (Southeast Atlantic; Latitude ~15–40°S, Longitude ~1–10°E).

We found a statistically significant change in the PO distribution in SA around 30°S and below 4000 m depth between 125 ka and 115 ka (Fig. 3). Simulated PO concentrations suggest a strong contrast between the deep western SA and the deep eastern SA in the distribution of NCW versus SCW from 125 ka to 115 ka (Fig. 3). At 125 ka, NCW has a predominant control over SCW in the deep western SA (Fig. 3c), whereas SCW dominates in the deep eastern SA (Fig. 3d), and the opposite is true at 115 ka. Such transition also induces changes in the deep water carbonate concentration below 4000 m due to the differing carbonate saturation levels of NCW and SCW respectively. In the west SA, deep water is characterized by more carbonate-saturated NCW at 125 ka than 115 ka, and vice versa in the east SA. This is also confirmed by the T-S plot of the deep waters in different regions in the Atlantic (Fig. 4), which supports that the deep water in the western SA switched from a primarily NCW origin at 125 ka to a mixture of NCW and SCW at 115 ka. Due to their northern positions these changes have not been recorded by the records obtained from ODP Site 1063 and MD03-2664 which imply a constant water mass distribution between both time slices.

At the end of the MIS5e (115 ka) higher PO values (Fig. 3) in the deep water below ~4000 m south of 30°S indicates a shoaling of NCW in the western SA. This region is therefore dominated by younger SCW formed in the SO (Fig. S2 in SI) at 115 ka while at 125 ka NCW reaches down below 4000 m as well.

**Figure 5:**
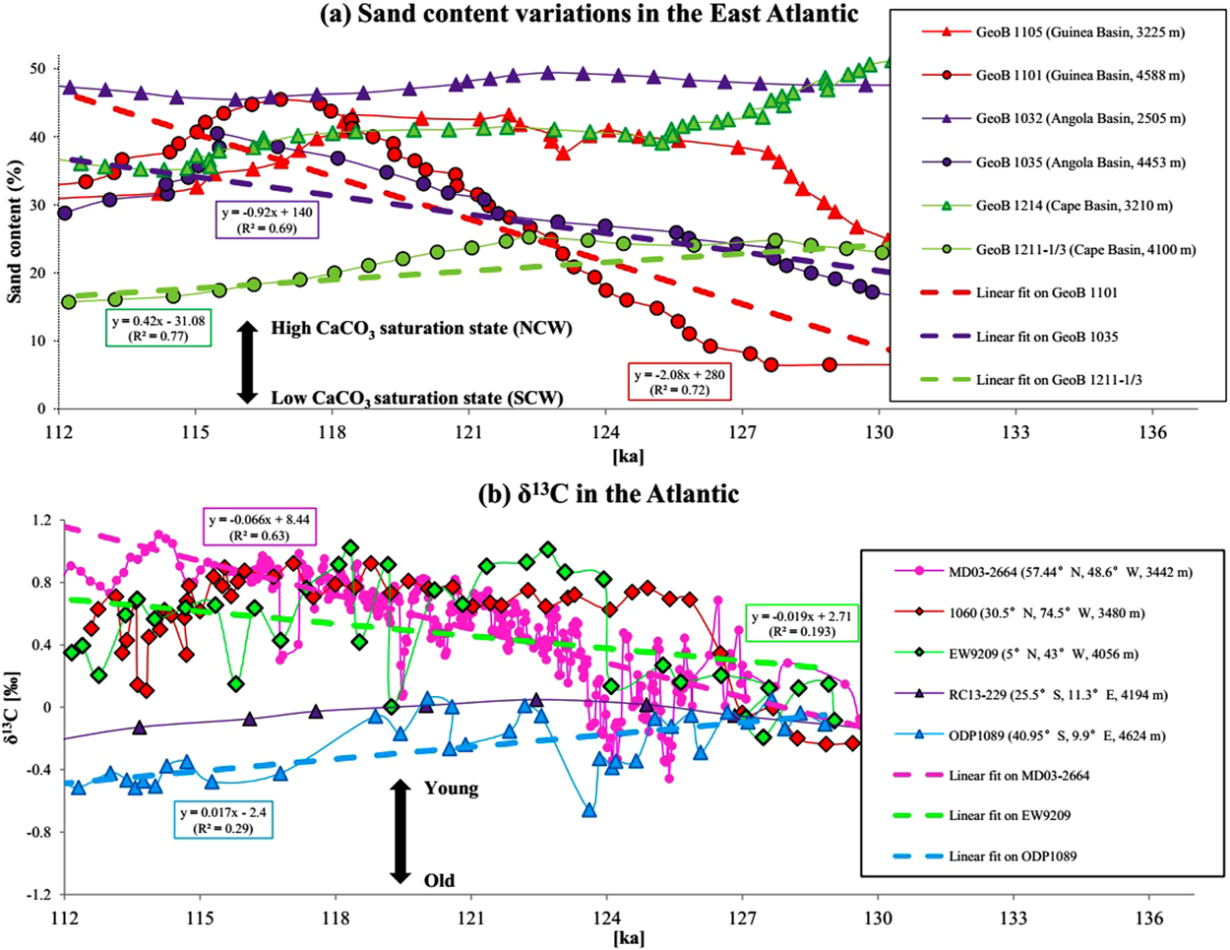
(**a**) sediment records for sand content variations in carbonates in six cores in the East Atlantic^30^ (core locations indicated in Figs 3 and 6; regressions are only shown for the data from sediments cores below 4000 m). The sediment data from above 4000 m are similar between 115 ka and 125 ka, and this is consistent with the model simulations, which suggest a west-east switch only for deep waters below 4000 m. (**b**) δ^13^C in five cores from the North Atlantic to the South Atlantic^23,24,33–36^. All records are shown on their original chronology.

Greater NCW influence in the deep eastern SA below about 4000 m at 115 ka, as indicated by carbonate concentrations from model simulations (Fig. 3), is consistent with sand content variations in the deep (>4000 m) eastern SA sediment cores^30^. Higher sand content in terms of particle size suggests reduced carbonate dissolution due to higher carbonate saturation state, and vice versa, because the sand content of deep-sea carbonates decreases as dissolution progresses^30^. The reason is that foraminiferal shells are affected by dissolution and tend to break down into small fragments. The abrupt increase in sediment sand content in the deep Guinea Basin (GeoB 1101, 4588 m, Fig. 5a) in the eastern SA from 125 ka to 115 ka, therefore, suggests a switch from more corrosive (less carbonate-saturated) SCW to less corrosive (more carbonate-saturated) NCW in this region A modest and similar transition of water mass change can also be found in the deep Angola Basin (GeoB 1035, 4453 m) sediment records, while the deep Cape Basin sediment (GeoB 1211, 4100 m) further south endures dissolution instead, consistent with intensified organic matter decay as a result of aging of the deep water south of ~30°S (Figs 5a, S2). However, sediment sand content records from depth above 3500 m in the Guinea Basin (GeoB 1105, 3225 m, Fig. 5a), the Angola Basin (GeoB 1032, 2505 m) and the Cape Basin (GeoB 1214, 3210 m) are relatively constant from 125 ka to 115 ka. This is also consistent with the model simulations, which suggest the water mass distributions at these shallower depth does not change significantly from 125 ka to 115 ka (Fig. 3).

**Figure 6:**
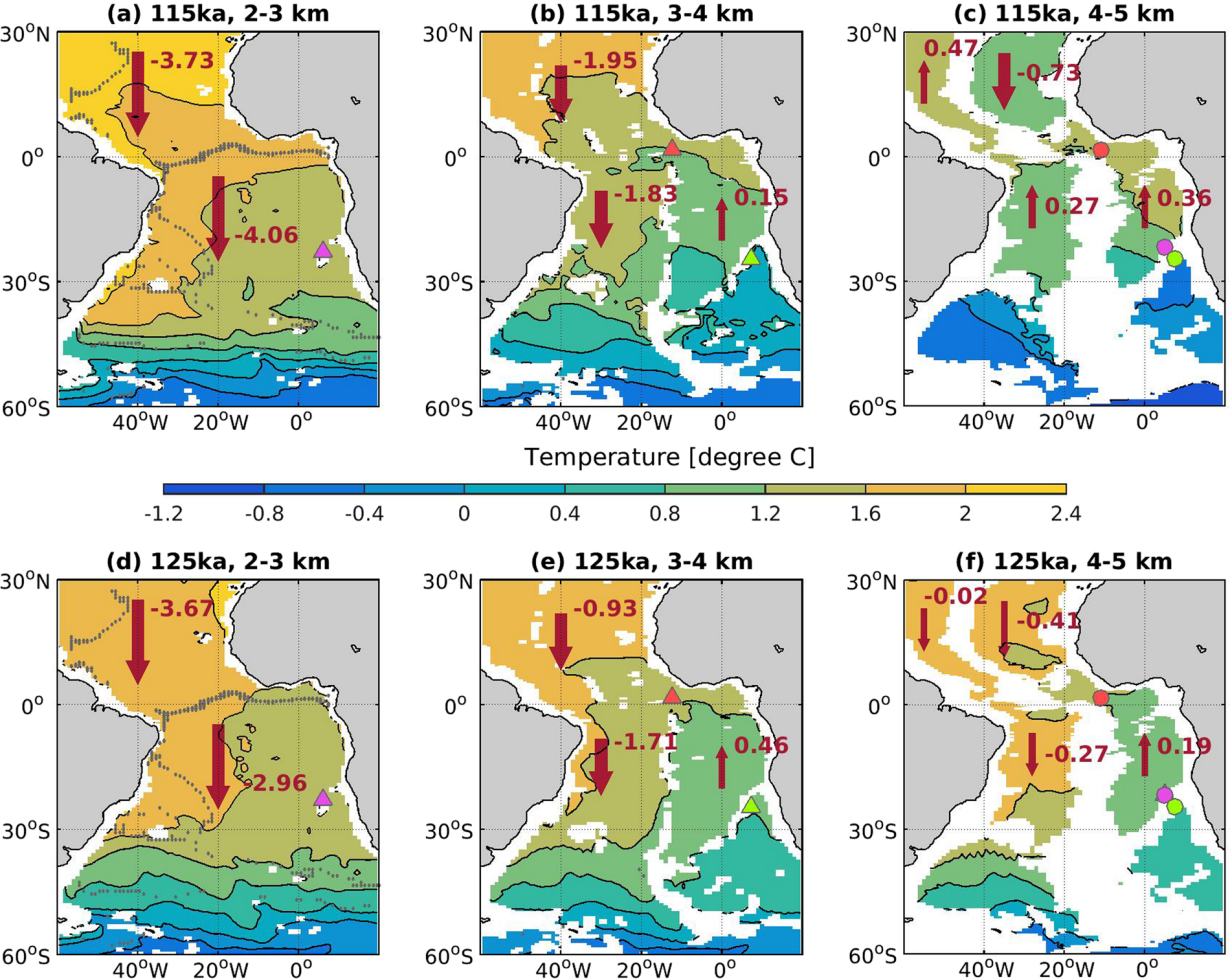
Water mass distributions indicated by temperature and deep water volume transport (Sv; positive value indicates northward direction and negative southward) in the South Atlantic for the 115 ka period between (**a**) 2–3 km, (**b**) 3–4 km, and (**c**) 4–5 km, and for the 125 ka period between (**d**) 2–3 km, (**e**) 3–4 km, and (**f**) 4–5 km. Markers indicate locations of the sediment cores for sand content in Fig. 5. The grids with stippling indicate h = 0 from the t-test, which suggest that the t-test does not reject the null hypothesis (results for 115 ka and 125 ka are statistically identical at the 5% significance level). Figure made with MATLAB R2014b.

In the NA, a shoaling of the Deep Western Boundary Current (DWBC) during the last interglacial has been suggested in response to a reduced presence of Lower NADW (LNADW)^31^. This change of water mass distributions is more difficult to observe in the SA, where mixing of more different water masses takes place. However, shoaled NADW at 115 ka results in a stronger control of the NCW at intermediate depths (e.g., between 2000 m and 3000 m) in the SA (Fig. 6a,d) but allows SCW to influence a larger volume in the deep SA basins below 4000 m (Fig. 6e,f). On the other hand, colder sea surface temperatures (SST; −1.8 ± 0.2 °C for 115 ka versus −0.6 ± 0.3 °C for 125 ka in the Weddell Sea region) in the Atlantic-sector SO at 115 ka lead to the formation of denser AABW at 115 ka than that at 125 ka (Figs 4, S3). Moreover, our model simulation suggests slightly higher surface salinity in the SO (Fig. S4), due to the strengthened ice formation and brine rejection at 115 ka. The 115 ka simulation shows thicker and wider coverage of sea ice in the Atlantic and Indian sectors of Antarctica compared to 125 ka, as a consequence of less annual insolation in the southern high latitudes at 115 ka^32^. Thus, colder (−1.4 °C ± 0.2 °C at 115 ka versus −0.5 °C ± 0.1 °C at 125 ka at 4500 m in the Argentine Basin) and slightly more saline (34.75 ± 0.1 at 115 ka versus 34.6 ± 0.1 at 125 ka) SCW at 115ka expands in the deep SA south of 30°S (Figs 6, S3–5 and it preferentially fills the deep Brazil Basin below 4000 m in the western SA and makes the NCW enter the deep Guinea Basin and Angola Basin in the eastern SA (Fig. 6c). In fact, density increase of the Southern Ocean surface water at 115 ka as a result of colder SST is more pronounced in the Pacific sector of the Southern Ocean (Fig. S6), and results in the production of denser deep water in the Pacific sector of the Southern Ocean (Fig. S7). Increased density of the SCW in the deep Argentine Basin at 115 ka are therefore not only associated with denser AABW formed in the Atlantic sector of the Southern Ocean, but also with denser deep water in the Pacific sector of the Southern Ocean.

The variations of the deep water properties (denser AABW; Figs S3–7) seem to be the controlling factor for this switch, as the surface SO westerlies are relatively stable over 125~115 ka (Fig. S8), which indicate they would have limited influence on the upwelling/downwelling strengths of South Atlantic waters.

## Interpretation of proxy data from the entire Atlantic

Carbon isotope data (δ^13^C) from water depths between ~3500 and 4200 m at different locations in the Atlantic Ocean consistently support the shoaling of NCW by showing similar increasing trends from ~125 ka to ~115 ka, but the magnitude of δ^13^C increase gradually weakens from the northern NA^24^, via subtropical NA^33^ and Equatorial Atlantic^23^, to the southern SA^34^, where no increasing trend is observable anymore (Fig. 5b). Considering that sediment δ^13^C data are more reliable as indicators of water mass properties, rather than strength of the circulation^11^, lower δ^13^C at 125 ka in the NA tends to suggest stronger SCW influence than at ~115 ka. In addition to δ^13^C, neodymium isotopic ratios and ^231^Pa/^230^Th ratios from the central SA core GeoB 3808–6 (30.8°S, 14.7°W, 3212 m water depth) both firmly indicate increased NCW influence at this water depth from the early to the late LIG^13^, consistent with the δ^13^C data interpretation.

At deeper depths, ε_Nd_ from the subtropical NW Atlantic ODP Site 1063 indicates only a slightly reduced influence of the NCW between ~125 ka and ~115 ka^26^ in the bottom water layer, and both δ^13^C and ^231^Pa/^230^Th are stable, suggesting that the strength and component of the deep flow at the same location remains the same^22,26^ (Fig. 1). In the SA, the sediment δ^13^C data in the deep Cape Basin^35,36^ below ~4000 m show a decreasing trend from 125 ka to 115 ka, in contrast to data from sediments at shallower depth (Fig. 5b). This indicates that the deep Cape Basin endures enhanced SCW control at 115 ka, which is consistent with our model simulations (Fig. S2).

Given the complexity of interpreting sediment δ^13^C data^37^, based on our compilation of the available sedimentary data (Fig. 5b) and other proxies as discussed in this study, we argue that mid-depth Atlantic Ocean between ~3000 m and 4000 m was influenced more by NCW at 115 ka than at 125 ka. Our data interpretation entirely supports our simulated deep-water formation scenarios over 125 ka–115 ka, but future improvement in spatial resolution of δ^13^C data^38,39^, integrated with model simulation is necessary to reaffirm our findings.

## Summary

Based on simulations from NorESM, we found that, although the AMOC is largely unchanged between 125 ka and 115 ka, the Northern Component Water (NCW) migrates from the western basins at 125 ka to the eastern basins at 115 ka in the deep layers of the South Atlantic below 4000 m. Our model results suggest a relatively constant North Atlantic Deep Water (NADW) overturning cell (about 300 m shallower at 115 ka compared to 125 ka) and colder southern sea surface temperature at 115 ka, which promotes stronger influence of SCW in the deep water below 4000 m, and consequently NCW occupies the intermediate depth in the Atlantic at 115 ka, consistent with proxy records. In the deep SA below 4000 m, the NCW migrates from the western to the eastern basin, due to the expanded dense SCW control in the deep western SA. We suggest future research on the investigation of the role of transient surface temperature changes, and water mass reconstructions in both eastern and western basins based on model simulations and paleoceanographic data to provide a more comprehensive understanding of past AMOC changes. Whilst our analysis generally supports earlier studies^36^, it reiterates that the mechanistic change of large-scale circulation is complex and challenging to infer using the available, yet sparse, geological proxy records.

## Methods

### Descriptions and set-up of last interglacial simulations

The last interglacial experiments presented in this study were performed with an updated version of the NorESM^20^. The current version applies a computationally efficient configuration that allows for multi-millennial and ensemble simulations. The model employs a two degree atmosphere/land grid, and a one degree ocean/sea ice horizontal resolutions. A 2000-year long pre-industrial experiment was performed and evaluated. The model skill in simulating pre-industrial climates is broadly similar with the original version with some improvements, especially in the representation of sea ice and AMOC. A comprehensive description and evaluation of the original NorESM has been documented in earlier studies^20,40^.

The two last interglacial experiments (125 and 115 ka BP) were branched off from year 1000 of the pre-industrial spin-up and were run for 1000 years with the respective boundary conditions (Table S1) for each time slice^41^. Experimental configurations follow the standard protocols of the third phase of Paleoclimate Modeling Intercomparison Project (PMIP3; https://pmip3.lsce.ipsl.fr/). Compared with the pre-industrial control experiment, only orbital parameters and concentrations of greenhouse gases are changed, whereas vegetation, ice sheet, topography, land/sea mask, and ocean bathymetry are kept the same as modern day. The atmospheric CO_2_ levels are set to 276 and 273 ppmv for 125 and 115 ka BP, respectively, compared to 284.7 ppmv in the pre-industrial experiment. The performance of our model has been tested in multi-model comparisons for paleoclimate/ocean reconstructions of different past time periods^41,42^. The last interglacial experiments are close to equilibrium after 1000 years, with very small trends in the top of atmosphere (TOA) radiation imbalance and global mean ocean temperature.

### Limitations

Model validation against proxy data demonstrates that last interglacial climates were reasonably simulated by the model, by showing a congruent contrast between 115 ka and 125 ka exhibited in the proxy-derived observations and the model results (e.g., see Fig. S9 for SST validation). However, a complete agreement between the model simulation results and the data is still not that clear, which needs to be improved in future research.

Fresh water perturbations are a well-known and well-identified reason for AMOC disturbances during the last deglacial. While the effect of melt-water on the AMOC is quantified by models and there is paleoceanographic evidence from proxy data on AMOC reduction, there is insufficient knowledge on the temporal and spatial distribution of melt-water fluxes and, even worse, on the magnitude of fresh-water fluxes. Reconstructions partly differ by a magnitude in the flux and do not give coherent timing^43–49^. The situation is, of course, even less constrained for the time around the Eemian. Given that there are no up-to-date reliable information on fresh-water pulses around these time periods we prefer to document the effects on AMOC driven the global boundary conditions rather than punctual and temporary fresh-water pulses.

We note that the simulations are from a single model, and given internal model uncertainty, future multi-model study would be useful to address the robustness of our findings.

## Electronic supplementary material

Supplementary Information
