# Supplementary material for: Atlantic deep water circulation during the last interglacial
Source: Sci Rep. 2018 Mar 13;8:4401. doi: 10.1038/s41598-018-22534-z (PMC5849616; doi:10.1038/s41598-018-22534-z)
Supplement: Supplementary file 1 — Supplementary Information [file 41598_2018_22534_MOESM1_ESM.pdf]

Supplementary Information for

"Atlantic deep water circulation during the last interglacial"

Yiming Luo[1], Jerry Tjiputra[2], Chuncheng Guo[2], Zhongshi Zhang[2], Jörg Lippold[3]

1. Geophysical Institute, University of Bergen and Bjerknes Centre for Climate Research, Allegaten 70, 5020 Bergen, Norway
2. Uni Research Climate, and Bjerknes Centre for Climate Research, Jahnebakken 5, 5007 Bergen, Norway
3. Institute of Earth Sciences, Heidelberg University, Im Neuenheimer Feld 234, 69120 Heidelberg, Germany

This supplementary information consists of Table S1-S2 and Figures S1-S11, which help to explain our model forcings, data compilation and the water mass distributions in the Atlantic Ocean.

Table S1, parameters for boundary conditions used for different scenarios. PI – Preindustrial.

Parameters		PI	115ka	120ka	125ka	130ka
Orbital parameters	ecc	0.0167	0.0414	0.0411	0.0400	0.0382
	obl	23.446 °	22.405 °	23. 012 °	23.798 °	24.242 °
	peri-180 °	102.04 °	110.88 °	27. 97 °	307.14 °	228.32 °
Date of vernal equinox		March 21 at noon	March 21 at noon	March 21 at noon	March 21 at noon	March 21 at noon
Trace gases	CO_2	284.7 ppm	273 ppm	269 ppm	276 ppm	257 ppm
	CH_4	791.6 ppb	472 ppb	573 ppb	640 ppb	512 ppb
	N_2O	275.7 ppb	251 ppb	262 ppb	263 ppb	239 ppb
	CFC	12.5 ppt	0	0	0	0
	O_3	Modern - 10 DU	Same as PI	Same as PI	Same as PI	Same as PI
Aerosols		Pre-industrial fixed	Same as PI	Same as PI	Same as PI	Same as PI
Solar constant		1361W/m^2	1365 W/m^2	1365 W/m^2	1365 W/m^2	1365 W/m^2
Vegetation		Pre-industrial fixed	Pre-industrial fixed	Pre-industrial fixed	Pre-industrial fixed	Pre-industrial fixed
Ice sheets		Present day	Same as PI	Same as PI	Same as PI	Same as PI
Topography and coastlines		Present day	Same as PI	Same as PI	Same as PI	Same as PI

Table S2, Information about the sediment cores and proxy used in the paper and their references.

Coring Site	Data Type	Latitude	Longitude	Water depth	Reference	Figure
ODP Site 1063	$\delta^{13}C$	33.69 °N	57.62 °W	4,584 m	Ref. 26	1
	εNd				Ref. 22	1
	$^{231}Pa/^{230}Th$				Ref. 22	1
MD03-2664	$\delta^{13}C$	57.44 °N	48.61 °W	3,442 m	Ref. 24	1, 5
1060	$\delta^{13}C$	30.5 °N	74.5 °W	3,480 m	Refs. 31, 33	5
RC13-229	$\delta^{13}C$	25.49 °S	11.31 °E	4,194 m	Refs. 34, 35	5
ODP1089	$\delta^{13}C$	40.95 °S	9.9 °E	4,624 m	Ref. 36	5
EW9209-1JPC	$\delta^{13}C$	5 °N	43 °W	4,056 m	Ref. 23	5
GeoB 1032-2/3	Sand Content	22.92 °S	06.04 °E	2,505 m	Ref. 30	5
GeoB 1035-3/4	Sand Content	21.59 °S	05.03 °E	4,453 m	Ref. 30	5
GeoB 1101-4/5	Sand Content	1.66 °N	10.98 °W	4,588 m	Ref. 30	5
GeoB 1105-3/4	Sand Content	1.66 °S	12.43 °W	3,225 m	Ref. 30	5
GeoB 1214-211	Sand Content	24.69 °S	07.24 °E	3,210 m	Ref. 30	5
GeoB 1211-1/3	Sand Content	24.48 °S	7.53 °E	4,100 m	Ref. 30	5

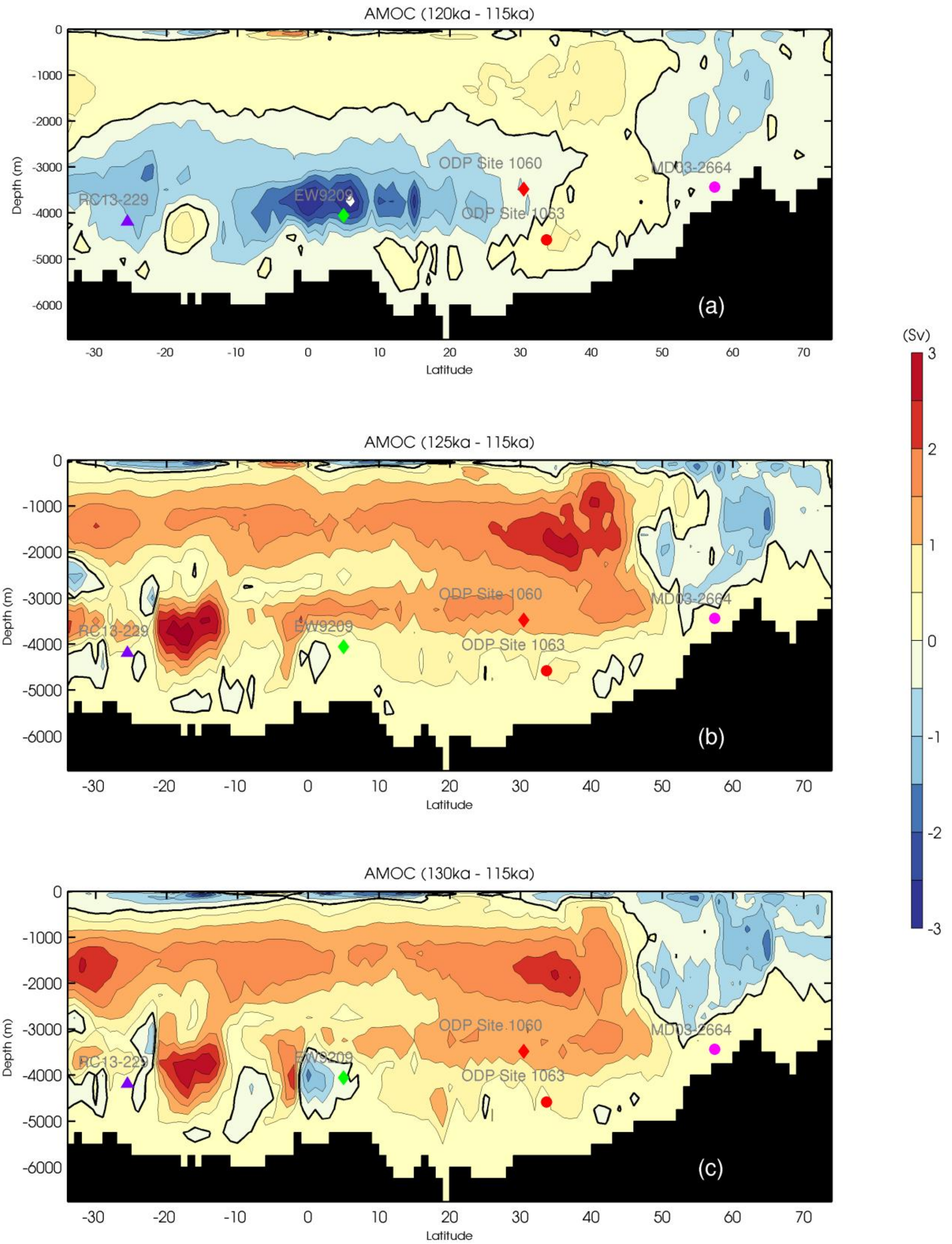

Figure S1: Difference of simulated AMOC strength between: (a) 120 ka and 115ka, (a) 125 ka and 115ka, (a) 130 ka and 115ka. Symbols indicate positions of sediment cores from which proxies of paleo-circulation are available for this time period. Figure made with MATLAB R2014b.

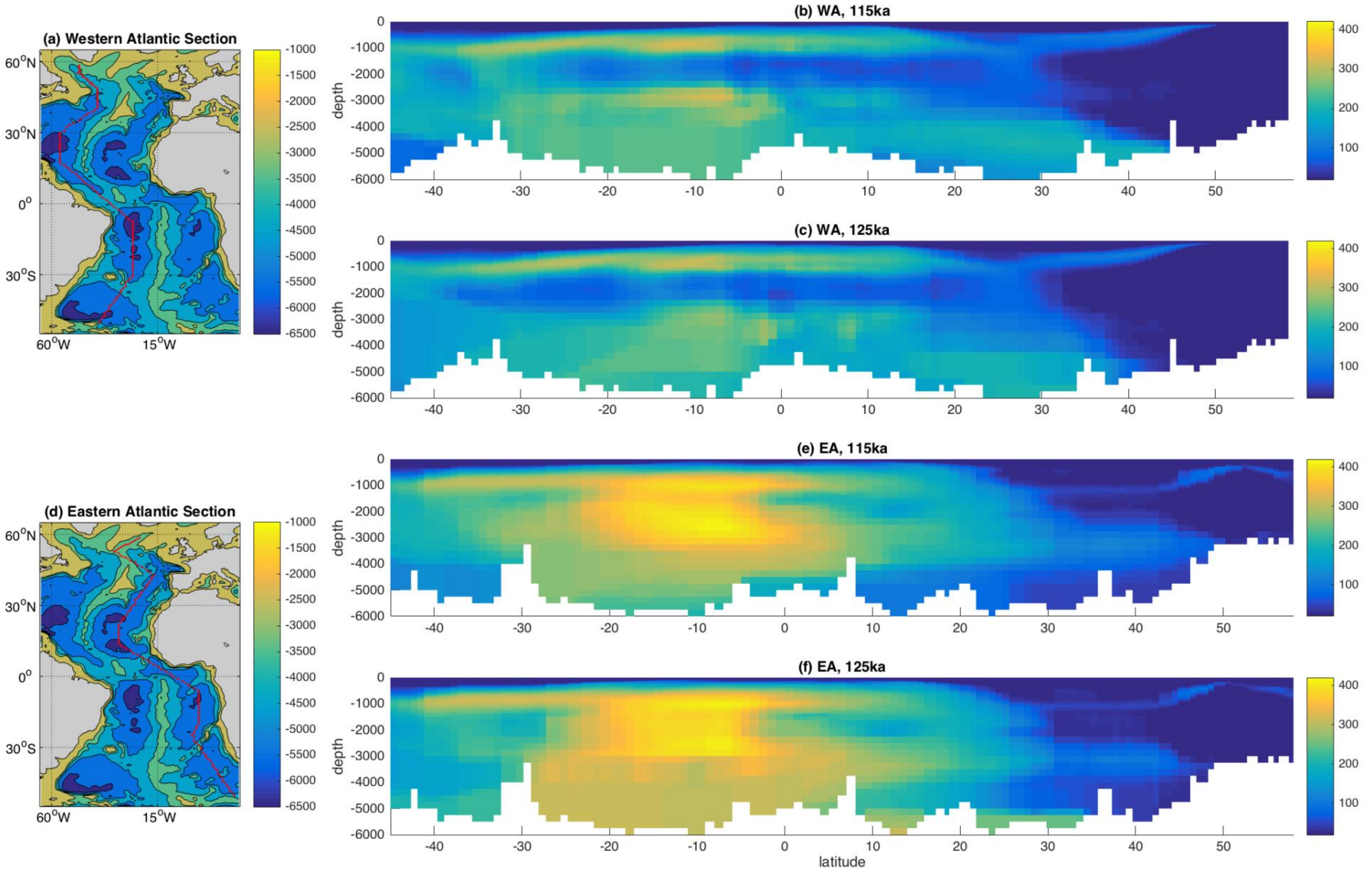

Figure S2: Ideal water mass ages (year) in the Atlantic Ocean: (a) the transect in the WA, (b) water mass profiles along the transect (a) in WA at 115ka, (c) water mass profiles along the transect (a) in WA at 125ka, (d) the transect in the EA, (e) water mass profiles along the transect (d) in EA at 115ka, (f) water mass profiles along the transect (d) in EA at 125ka. Figure made with MATLAB R2014b

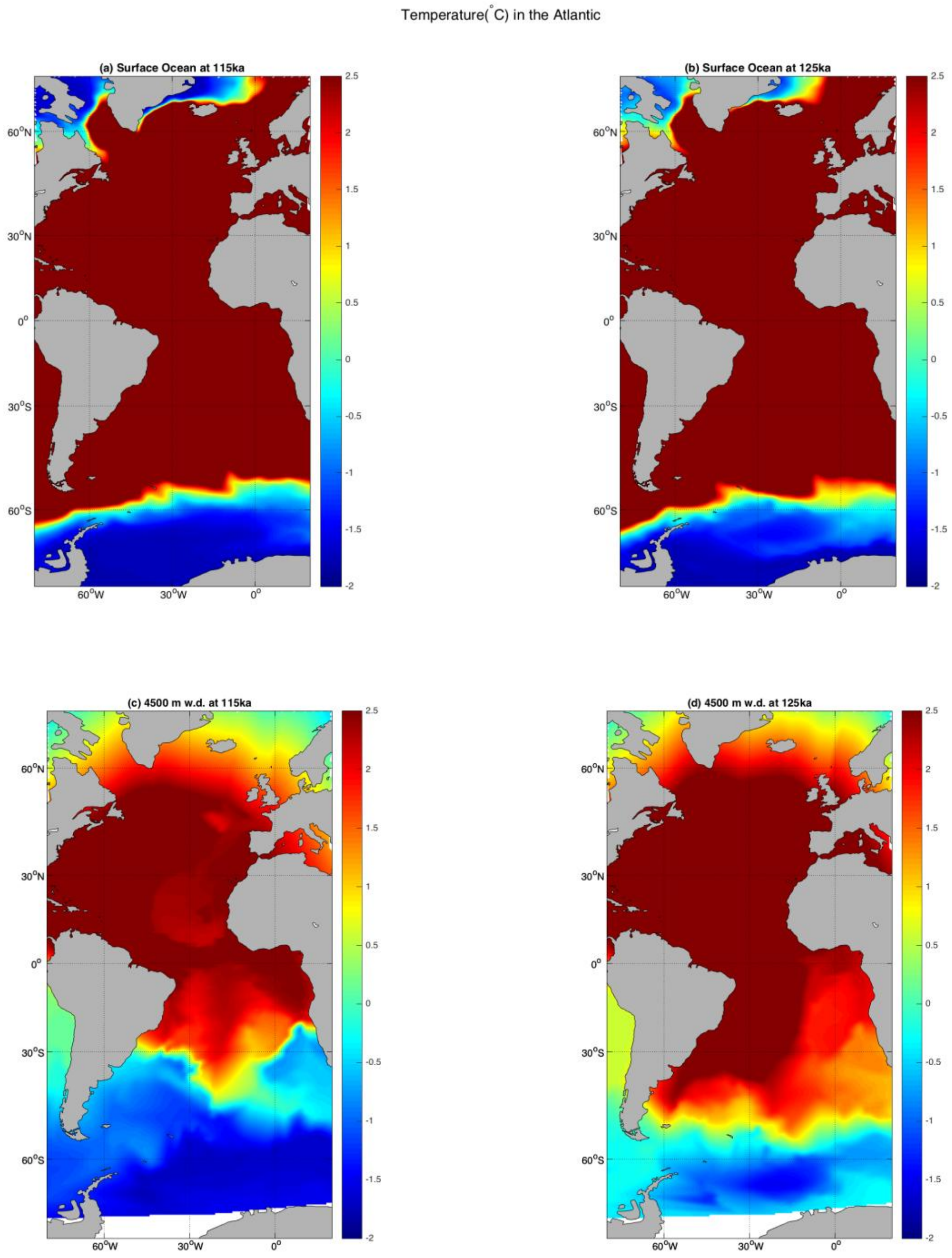

Figure S3: Model simulated temperature distributions in the Atlantic for: Surface Ocean (a) 115ka, (b) 125ka and Deep Ocean (c) 115ka and (d) 125ka at 4500 m. Figure made with MATLAB R2014b.

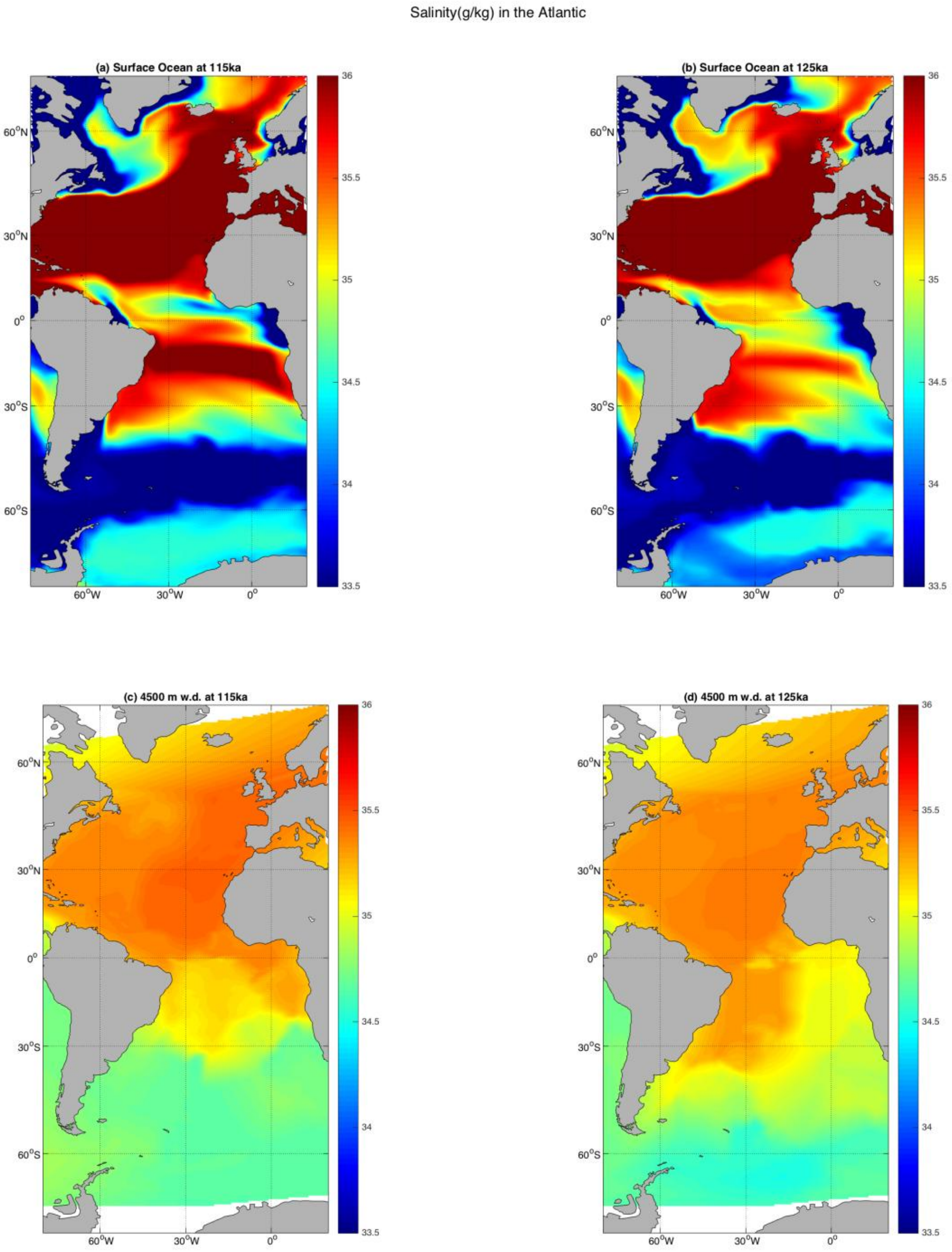

Figure S4: Model simulated salinity distributions in the Atlantic for: Surface Ocean (a) 115ka, (b) 125ka and Deep Ocean (c) 115ka and (d) 125ka at 4500 m. Figure made with MATLAB R2014b.

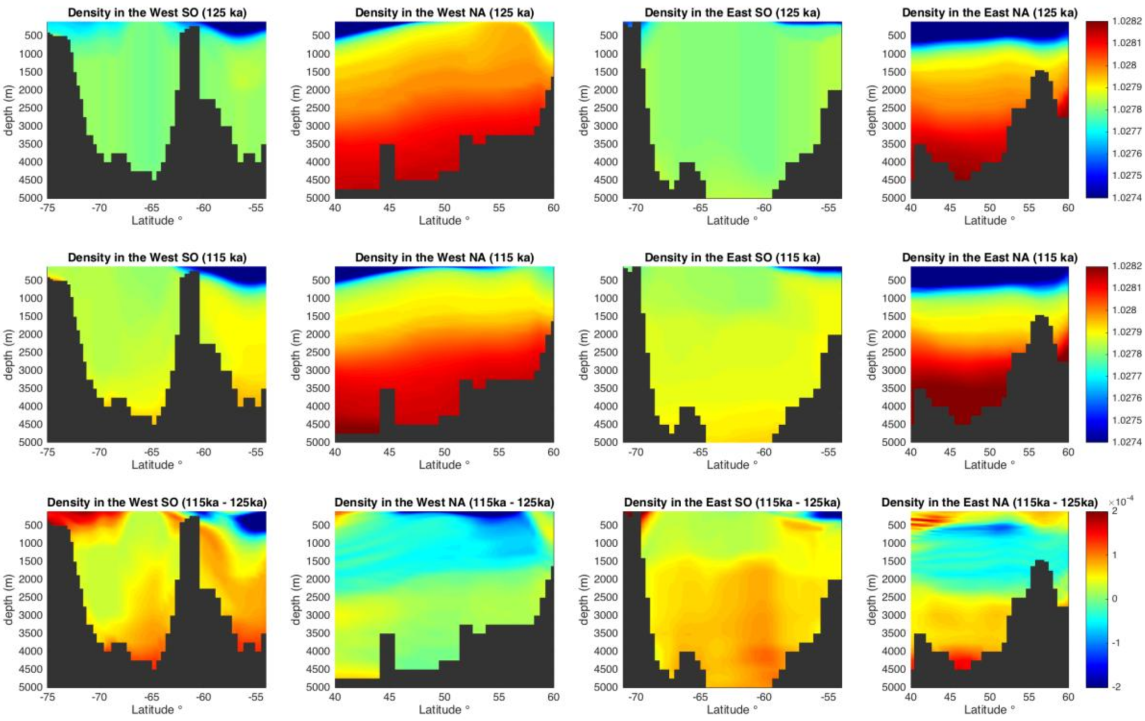

Figure S5: Model simulated water density in different regions for: 125 ka (the first row), 115 ka (the second row) and the difference between the 115ka and 125 ka (the third row). Figure made with MATLAB R2014b.

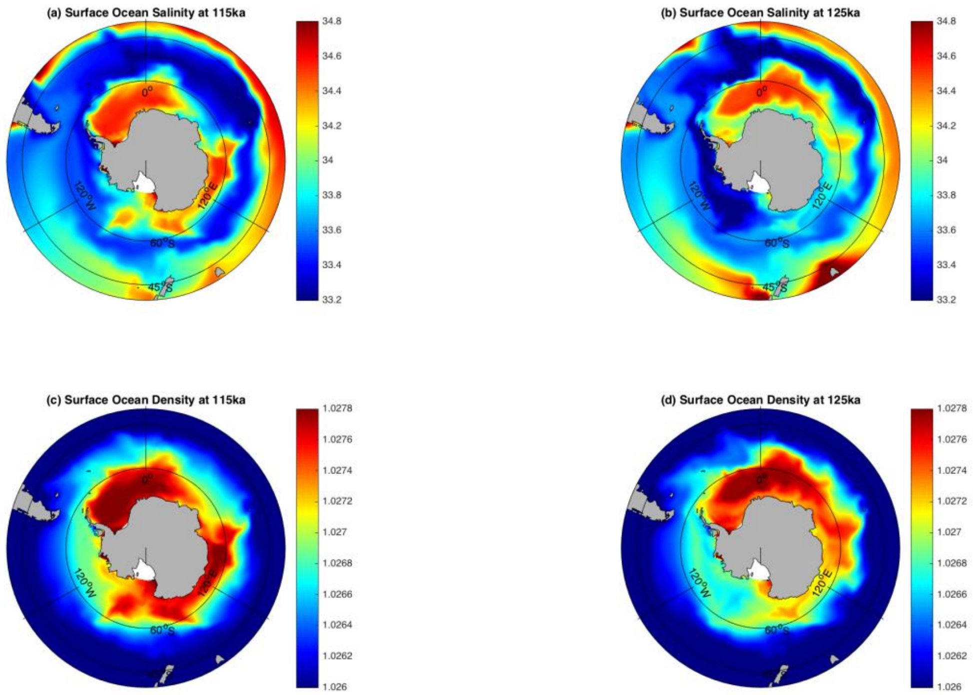

Figure S6: Model simulated surface ocean water density in the Southern Ocean for: 115 ka (a), 125 ka (b) and surface ocean sea water density in the Southern Ocean for: 115 ka (c), 125 ka (d). Figure made with MATLAB R2014b.

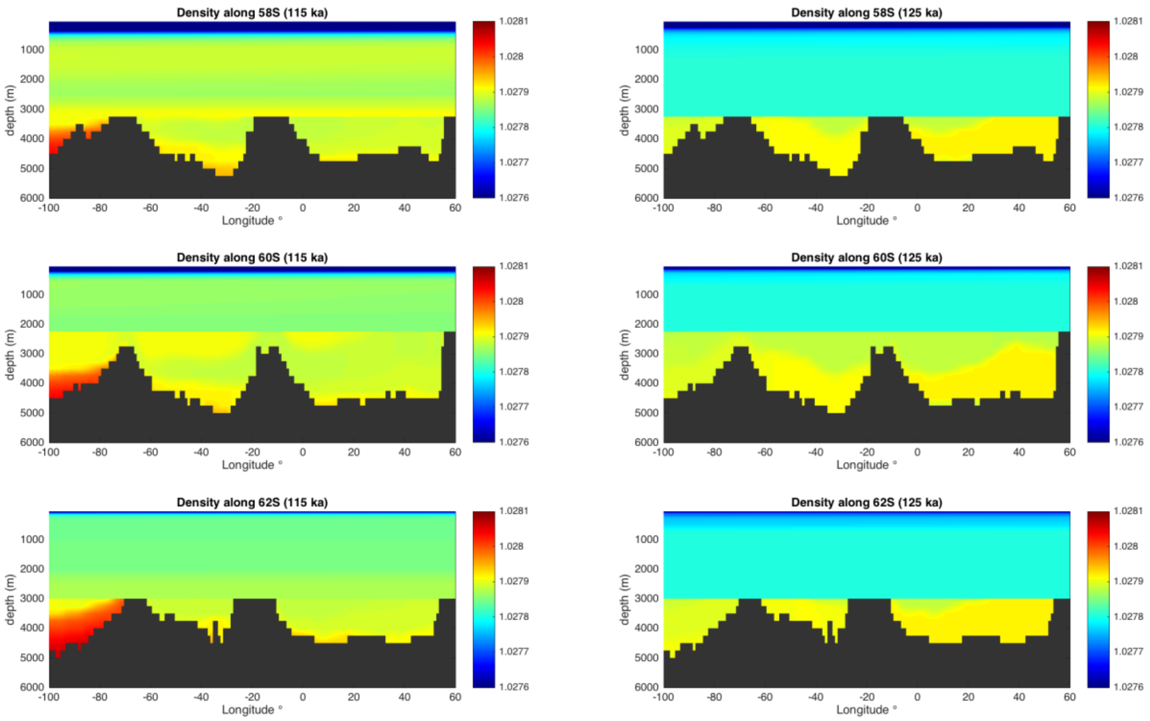

Figure S7: Model simulated water density in the Atlantic sector of the Southern Ocean for 115 ka along (a) 58 °S, (b) 60 °S and (c) 62 °S and for 125 ka along (d) 58 °S, (e) 60 °S and (f) 62 °S. Figure made with MATLAB R2014b.

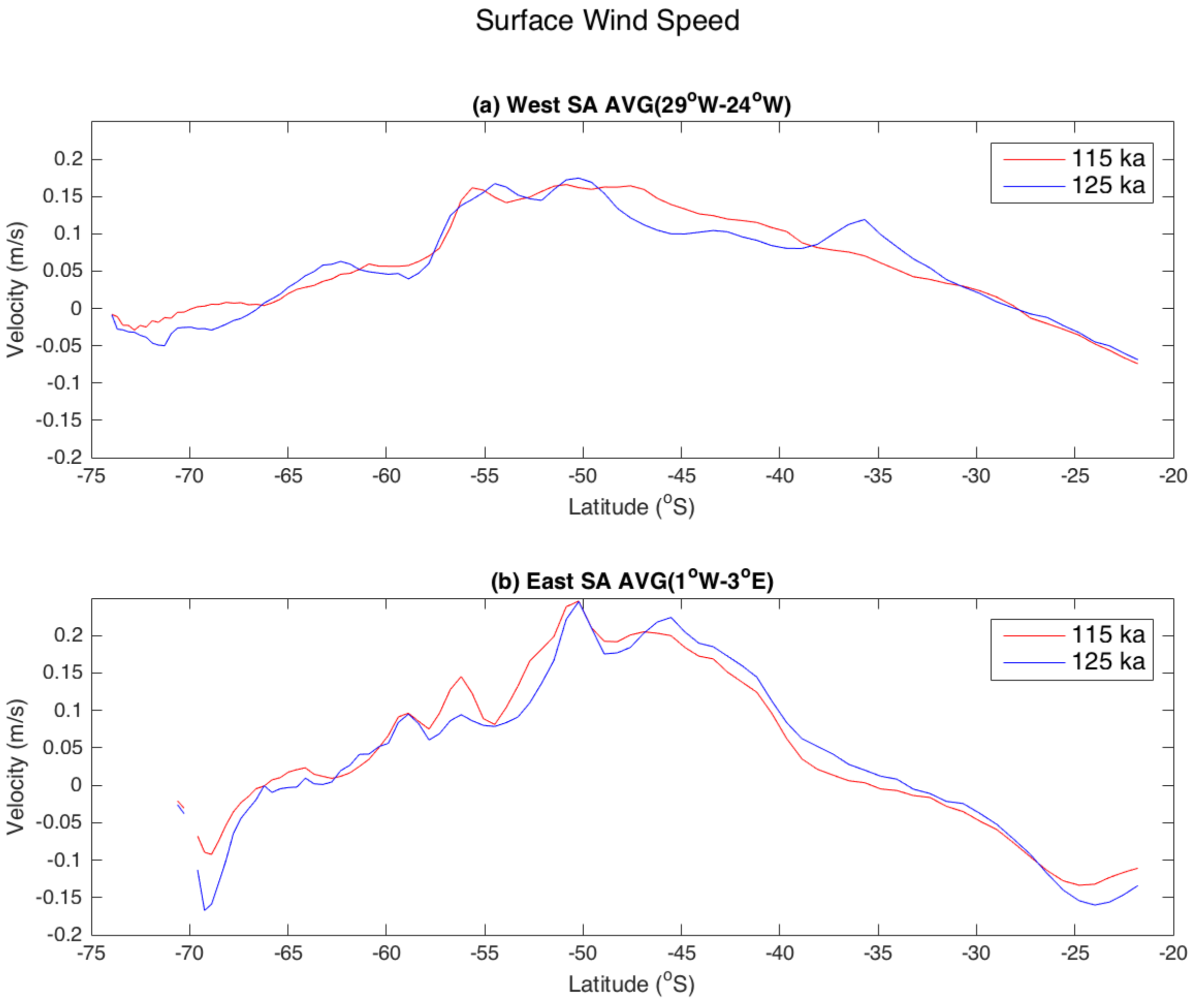

Figure S8: Simulated strength of the surface westerlies in the South Atlantic in: (a) west SA (average for 29 °W - 24 °W), (b) east SA (average for 1 °W - 3 °E). (Positive velocity indicates eastward wind direction and negative westward)

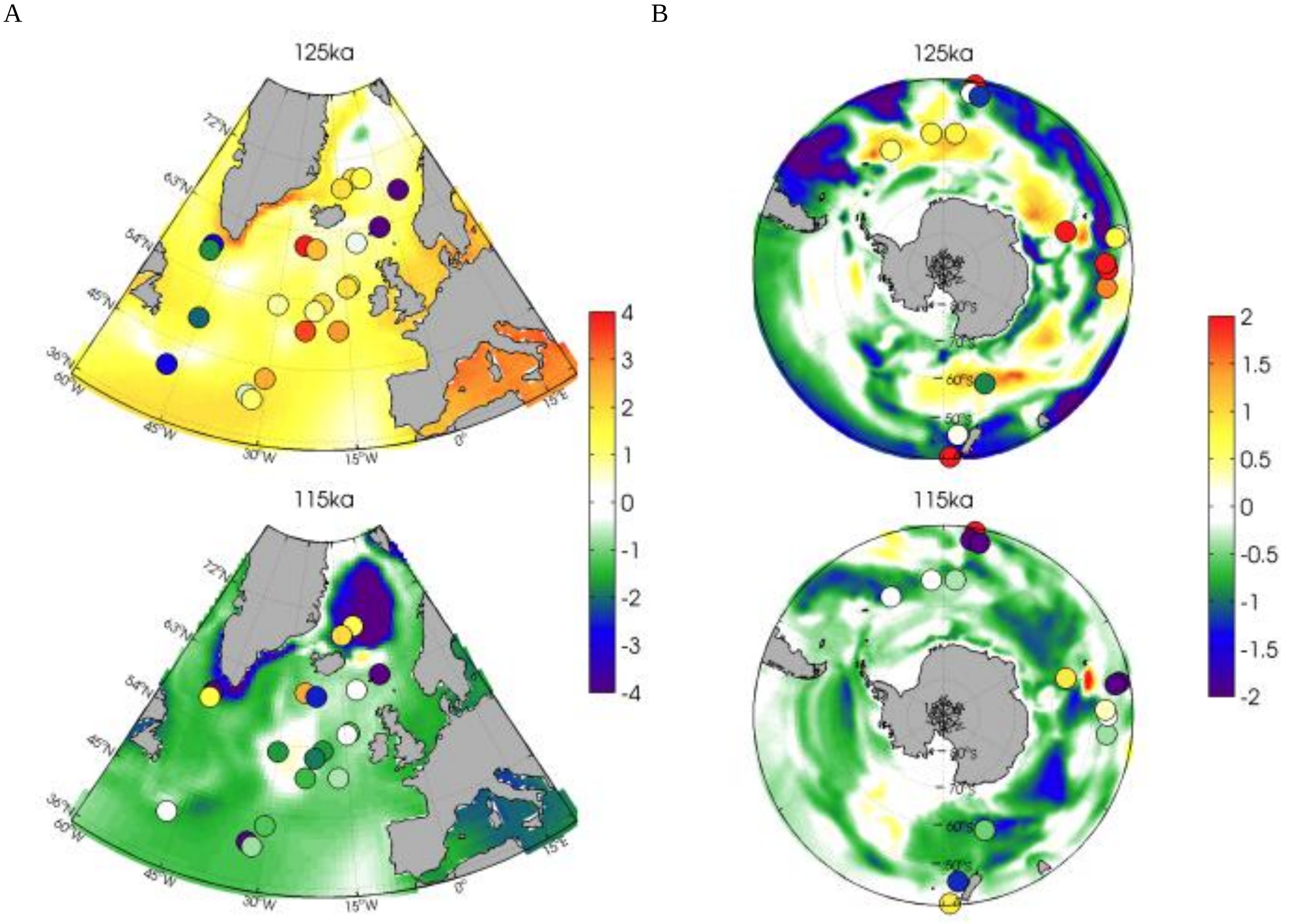

Figure S9: Model validation: summer SST simulation versus proxy (Capron et al., 2014) for 115ka and 125ka for the North Atlantic (A) and Southern Ocean (B) regions. While a complete agreement with the data is not that clear, there is a congruent contrast between 115ka and 125ka, exhibited in the proxy-derived observations and the model results. Figure made with MATLAB R2014b.

Reference:

Capron, E. et al. Temporal and spatial structure of multi-millennial temperature changes at high latitudes during the Last Interglacial. *Quat. Sci. Rev.* **103**, 116-133 (2014).
